# Harbor and Intra-City Drivers of Air Pollution: Findings from a Land Use Regression Model, Durban, South Africa

**DOI:** 10.3390/ijerph17155406

**Published:** 2020-07-27

**Authors:** Hasheel Tularam, Lisa F. Ramsay, Sheena Muttoo, Rajen N. Naidoo, Bert Brunekreef, Kees Meliefste, Kees de Hoogh

**Affiliations:** 1 Discipline of Occupational and Environmental Health, University of KwaZulu-Natal, Durban 4041, South Africa; ramsayl@ukzn.ac.za (L.F.R.); sheena.muttoo@gmail.com (S.M.); naidoon@ukzn.ac.za (R.N.N.); 2 Institute for Risk Assessment Sciences, Utrecht University, 3508TD Utrecht, The Netherlands; B.Brunekreef@uu.nl (B.B.); C.Meliefste@uu.nl (K.M.); 3 Department of Epidemiology and Public Health, Swiss Tropical and Public Health Institute (Swiss TPH), Socinstrasse 57, CH-4002 Basel, Switzerland; c.dehoogh@swisstph.ch; 4 Faculty of Science, University of Basel, CH-4003 Basel, Switzerland

**Keywords:** exposure assessment, land use regression, ship emissions, air pollution monitoring

## Abstract

Multiple land use regression models (LUR) were developed for different air pollutants to characterize exposure, in the Durban metropolitan area, South Africa. Based on the European Study of Cohorts for Air Pollution Effects (ESCAPE) methodology, concentrations of particulate matter (PM_10_ and PM_2.5_), sulphur dioxide (SO_2_), and nitrogen dioxide (NO_2_) were measured over a 1-year period, at 41 sites, with Ogawa Badges and 21 sites with PM Monitors. Sampling was undertaken in two regions of the city of Durban, South Africa, one with high levels of heavy industry as well as a harbor, and the other small-scale business activity. Air pollution concentrations showed a clear seasonal trend with higher concentrations being measured during winter (25.8, 4.2, 50.4, and 20.9 µg/m^3^ for NO_2_, SO_2_, PM_10_, and PM_2.5_, respectively) as compared to summer (10.5, 2.8, 20.5, and 8.5 µg/m^3^ for NO_2_, SO_2_, PM_10_, and PM_2.5_, respectively). Furthermore, higher levels of NO_2_ and SO_2_ were measured in south Durban as compared to north Durban as these are industrial related pollutants, while higher levels of PM were measured in north Durban as compared to south Durban and can be attributed to either traffic or domestic fuel burning. The LUR NO_2_ models for annual, summer, and winter explained 56%, 41%, and 63% of the variance with elevation, traffic, population, and Harbor being identified as important predictors. The SO_2_ models were less robust with lower R^2^ annual (37%), summer (46%), and winter (46%) with industrial and traffic variables being important predictors. The R^2^ for PM_10_ models ranged from 52% to 80% while for PM_2.5_ models this range was 61–76% with traffic, elevation, population, and urban land use type emerging as predictor variables. While these results demonstrate the influence of industrial and traffic emissions on air pollution concentrations, our study highlighted the importance of a Harbor variable, which may serve as a proxy for NO_2_ concentrations suggesting the presence of not only ship emissions, but also other sources such as heavy duty motor vehicles associated with the port activities.

## 1. Introduction

Quantifying an individual’s exposure to air borne pollutants remains a key challenge in epidemiological studies as the level of exposure depends on both the spatial-temporal dynamics of air pollution concentrations and the individual’s activities. Each individual has their own unique personal exposure to air pollution during their daily life, occurring both in indoor and outdoor environments, and therefore the quantifying process is complex [1]. To determine the effect of these exposures on health, many of these studies have estimated individual air pollution exposure by making use of air quality monitoring datasets that are representative of the study area and have also made use of more complex approaches such as spatial interpolation [2,3,4].

Proximity-based estimates, interpolation methods (e.g., kriging and inverse distance weighting) and more complex atmospheric dispersion models are methods that are commonly being used to undertake spatial exposure assessments. Proximity-based estimates give some indication of health impact; however, these do not adequately consider meteorology or source characteristics and can therefore result in exposure misclassification [5]. Kriging, as an example of a spatial interpolation method, has been found to be increasingly effective when being applied to regional or national scale and not at a local scale [6,7]. Dispersion models are able to provide a higher level of accuracy at the local scale however input data demands and specialized expertise limit its application [5,8].

As a result, further developing exposure models for epidemiological purposes remains imperative as these models can typically be used to supplement monitoring datasets where direct measurements are not available as well as assist by reducing expensive and resource intensive monitoring programs. Furthermore, the contribution of different air pollutant chemicals can be clearly separated in an exposure assessment to determine their health effects.

Air pollution exposure models can be used in health risk assessments, for effectively siting ambient air quality monitoring networks, and for developing air quality related policies and management plans. Air quality monitoring instrumentation with high precision, accuracy, and temporal resolution is costly to deploy. While data obtained from monitoring stations are a useful tool for exposure assessments, these fixed sites of measurements do not show geographical variations in pollutant dispersion, which is essential for calculating individual impact. Land use regression (LUR) modelling is an alternative to these approaches, allowing for the calculation of air pollution concentrations at a high spatial resolution without requiring a detailed air pollution emissions inventory [3]

LUR combines the monitoring of air pollution at a number of locations with stochastic modelling using predictor variables obtained through Geographic Information Systems (GIS). Typical examples of geographic predictor variables include land use type, population, traffic intensity, topography, and meteorology. Issues regarding the availability of, or access to, complete and reliable predictor data (i.e., traffic variables, population or housing density, land use, altitude, topography, meteorology, and location) can hinder LUR studies, but generally these models offer a reliable alternative to more complex dispersion models that require detailed meteorological data inputs.

Published LUR models have been developed for sites in Europe, North America, and Japan [9,10,11,12,13]. Since nitrogen dioxide (NO_2_) has previously shown to correlate well with traffic densities in numerous LUR assessments, this pollutant has been used as a proxy for traffic emissions. Some LUR studies have even investigated the importance of incorporating meteorological variables for predicting air pollutant concentrations [14,15].

An example of multisite LUR model development and its application is the European Study of Cohorts for Air Pollution Effects (ESCAPE). The ESCAPE study developed LUR models to estimate exposure at the residential addresses of cohort participants based on uniform monitoring campaigns and uniform modelling approaches in 36 study areas located all over Europe [16,17,18,19]. Furthermore, within the ESCAPE framework, LUR models have been successfully developed to estimate the spatial variation of annual mean concentrations for various pollutants including PM [19], elemental composition [18], nitrogen dioxide (NO_2_), and nitrogen oxides (NO_x_) [16]. Since these models were used extensively to assess the association between long-term exposure to air pollution and specific health outcomes, it was selected for application in this study.

This study aims to characterize the spatial distribution of nitrogen dioxide (NO_2_), sulphur dioxide (SO_2_), particulate matter with an aerodynamic diameter of less than 10 µm (PM_10_) and of less than 2.5 µm (PM_2.5_) concentrations in Durban, South Africa, accounting for surrounding land use variables, e.g., land use type and traffic intensity. We addressed this through the application of the ESCAPE methodology.

### Study Background

**Figure 1:**
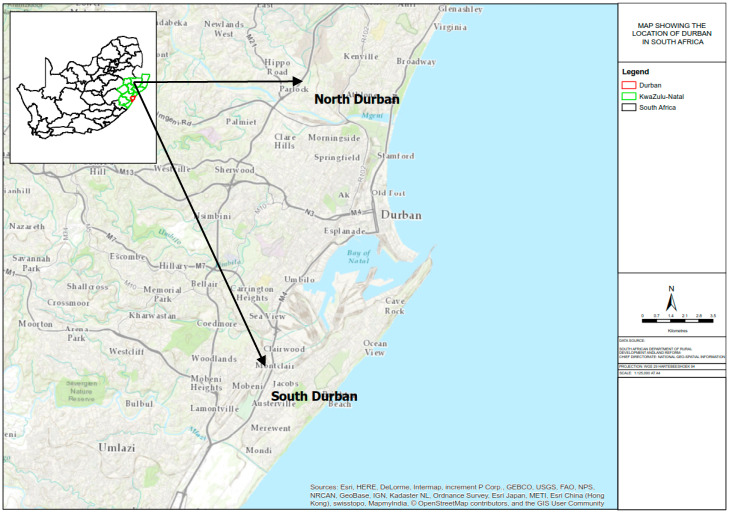
Location of Durban north and Durban south, and their location within South Africa (inset map).

Durban is located within the eThekwini Metropolitan Municipality on the east coast of South Africa. The municipality is home to some 3.5 million people and extends approximately 50 km southwest, 35 km northeast, and 45 km west of the central business district CBD, spanning an area of approximately 2297 km^2^ (Figure 1).

Air pollution in Durban results from a variety of activities. Apart from ship emissions, as it is the busiest port on the African continent, pollution sources include petrochemical refining, pulp and paper industries, metallurgical industries, organic chemical industries, to smaller facilities such as transportation, domestic fuel burning, landfills, and quarries [20]. These industries are regulated by the South African National Ambient Air Quality Standards, NAAQS (Government Notice 1210 of 2009) [21]. Air pollution sources ranging from large industrial facilities, qualify as regulated listed activities [22] South Durban is considered the economic hub of KwaZulu-Natal due to high density of industries within this district. North Durban, however, comprises primarily residential land with a limited light industrial activity.

The south Durban and north Durban areas formed the focus of this assessment (Figure 1). The “Durban South Industrial Basin” (DSIB) is a well-defined, narrow strip of land, approximately 5 km wide, extending south-westwards from the Durban Harbor for approximately 12 km. It covers an area of approximately 40 km^2^ and comprises a mixture of land use zones including industrial, residential, and commercial. Historically, individuals residing in the DSIB remain at high risk for exposure to significant levels of ambient air pollution due to their location to sources of air pollutants. Specifically, two major petroleum refineries, as well as a pulp and paper manufacturing plant are located within the community. The area is linked to other major urban centers via an extensive road network.

Since promulgation of the NEM: AQA in 2004, as well as development of the cities Air Quality Management Plan (AQMP) [23] in 2015, there has been an increased effort towards reducing air pollution to a level conducive to the health and wellbeing of people living in this area. More stringent Minimum Emission Standards (MES) [22] being applied in a staged approach over time, have allowed industries to modify their processes such that they reduce their air pollution impact in the area in which they operate [24]. The north Durban study area comprises the residential areas of Newlands, Kenville, Broadway, Virginia as well as light industrial business parks that have developed along the Umgeni River and along the R102 and Umgeni Road.

## 2. Materials and Methods

The ESCAPE methodology was generally applied in this LUR assessment of Durban, KwaZulu-Natal, South Africa. The methodology employs a mixture of air pollution monitoring and modelling techniques to estimate exposure at specific GIS locations to ambient air pollution, accounting for surrounding land use.

### 2.1. Monitoring Site Selection

**Figure 2:**
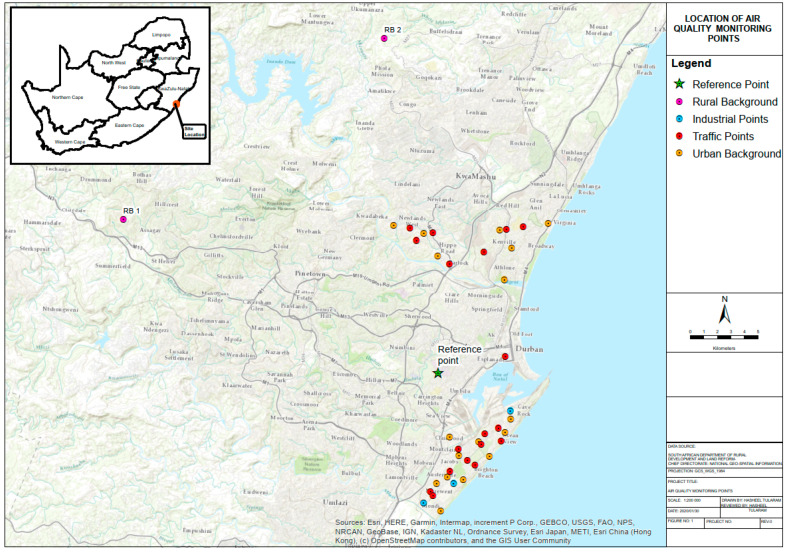
Location of air quality monitoring points in Durban north and Durban south, and their location within South Africa.

Monitoring sites were selected to best represent the spatial variation of air pollution in north Durban and South Durban. Regional background, urban sites, and street type-sites were identified (Figure 2). The urban sites were selected because they were not significantly affected by air pollution in their direct vicinity, with no more than 3000 vehicles per a day passing within 50 m of the site or other key air pollution sources (industries, combustion sources, etc.) present within a radius of 100 m. The street type-site represented traffic pollution and was defined as an area where traffic intensity exceeded 10,000 vehicles per a day [16]. The regional background sites were located away from local source activity to represent a long-term average of ambient pollutant concentrations.

The following criteria were adopted for specific site selection:

- Monitoring sites were not located within 25 m of a traffic intersection;
- Monitoring sites were at least 2 m from the roadside;
- Monitoring sites were not located with 100 m of construction activities; and
- Sampling points were selected such that airflow around the samplers were unrestricted by buildings.

### 2.2. Monitoring Equipment Installation

**Figure 3:**
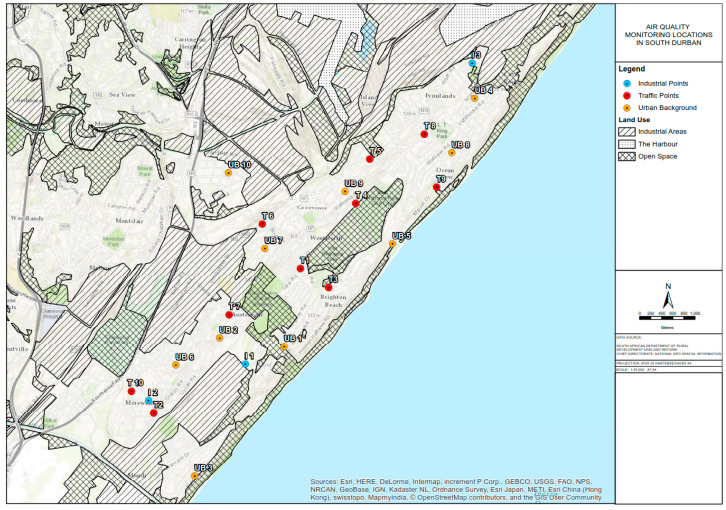
Location of air quality monitoring samplers in south Durban.

**Figure 4:**
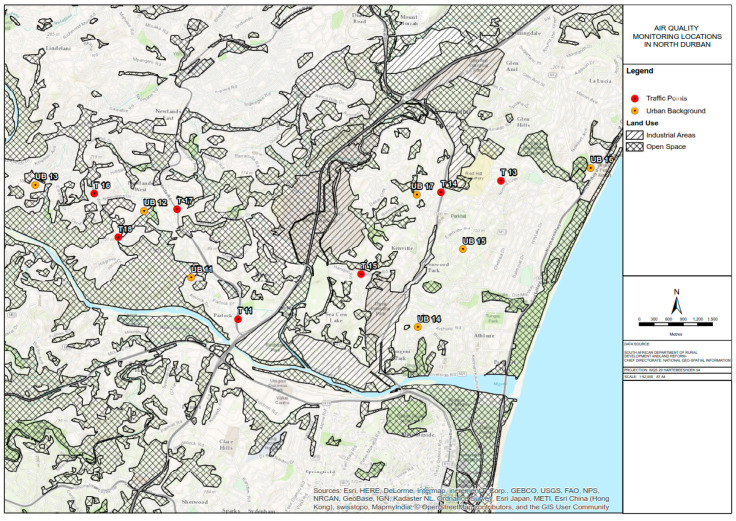
Location of air quality monitoring samplers in north Durban.

In this study, Ogawa passive samplers were used to measure NO_2_ and SO_2_ concentrations while Harvard impactors were used to measure PM_10_ and PM_2.5_ concentrations according to standard operating procedures (SOPs) adopted in other ESCAPE studies [25]. PM_10_ and PM_2.5_ measurements were conducted at 20 monitoring sites (11 in south Durban and nine in north Durban) while passive NO_2_ and SO_2_ measurements were conducted at 40 monitoring sites (23 in south Durban and 17 in north Durban) within the eThekwini Municipality (as shown in Figure 2, Figure 3 and Figure 4 below). Measurements were conducted over 2-week periods per a site with a 1-week break in between in the winter (June–August), spring (September–November 2015), and summer (December–February) seasons.

Since measurements were not performed continuously, at one additional site (the reference site), PM, NO_2_, and SO_2_ were measured using the same instruments for a period of 1 year (July 2015–June 2016). This allowed for sites that were only measured over the three seasons to be adjusted to the long-term average for the monitoring period. As such there were 21 PM monitoring sites and 41 NO_2_ and SO_2_ monitoring sites in total (Figure 3 and Figure 4). The reference site was selected at the University of KwaZulu-Natal as it is strategically located between north and south Durban where a bulk of the monitoring was undertaken. Furthermore, this location was easily accessible as this station required to be fully operational throughout a 1-year period. For quality control purposes, one field blank per a pollutant (PM, NO_2_, and SO_2_) was collected at the reference site such that 12 field blanks were collected over the sample period. The field blanks were used to calculate the limit of detection of the samples. Furthermore, one field duplicate (PM_10_, NO_2_, and SO_2_) was collected at the reference site for the measurement period. Field duplicates were logged to determine the accuracy of measured pollutant concentrations. Furthermore, a rotameter was used to measure the volumetric flow rate of each Harvard impactor at the start and end of each 2-week monitoring period. Further quality assurance details are provided in the Supplementary Materials.

The particle mass on each filter was determined by weighing the filter before and after field sampling. The pump units consist of a 10 L per a minute pump, two timers (a weekly and 24 h), and an elapsed time indicator. The total volume of air sampled was recorded using a built-in timing device. The pump unit is built into weatherproof case comprising of ventilators to allow the unit to cool should the temperature in the box get too high. The impactors were deployed such that the inlets were at a height of about 1.5 m above the ground.

Timers were used to allow the pump to operate for 15 min during every 2 h. All samples collected were stored at 4 °C. Post 24-h conditioning, all filters were pre and post weighed as per RUPIOH SOP version 3 weighing protocol. Samples were then shipped to University of Utrecht to test for reflectance and determine the absorption coefficient using a Smoke Stain Reflectometer: Diffusion Systems Ltd. Model 43 (M43D).

All Ogawa passive samplers (for NO_2_ and SO_2_) were deployed at 2 m above the ground at each sample point. Upon collection, samples were stored at 4 °C before being couriered to a South African National Accredited System (SANAS) laboratory for analysis.

To determine the annual average for each monitoring site, the results from each 2-week sampling period had to be adjusted using data from the reference site as monitoring was continuously undertaken at this point for a period of 1 year. The arithmetic means of the available measurements (i.e., both sampling periods) per site were adjusted using the difference between the sampling period and the annual average at the reference site thus deriving an annual adjusted average for each pollutant.

### 2.3. Geographic Predictor Variables

Important predictor variables as indicated by [9], included traffic, housing density, population density, land use type, physical geography, and meteorology. The eThekwini Municipality Corporate GIS Unit provided the geographical information for the study area. GIS shape files collected include roads, land use, population density, as well as physical geography such as altitude and distance to coastline. The land use data was divided into industrial, open space, urban, and Harbor.

Road linkages were categorized into two groups (major and minor) based on traffic intensity. The eThekwini Municipality Traffic Authority provided traffic count data for light duty motor vehicles (LDMV), and heavy-duty motor vehicles (HDMV) for major intersections along roads in south Durban and north Durban (period 2013–2017). While road length and distance to road classifications were also used to determine the effect of traffic on air pollution [3,9], traffic count data was also obtained. This served to further explore the effect of the number of HDMV and LDMV on the measured air pollutant concentrations in north Durban and south Durban.

Since wind speed and direction is regarded as a key meteorological parameter in the dispersion of air pollution, this study also investigated this phenomenon by assessing a wind trajectory in relation to industry location. Meteorological data in south Durban and north Durban was obtained from the South African Weather Services meteorological station located at the old Durban International Airport and Mount Edgecombe, respectively. Hourly wind speed, wind direction, ambient temperature, and humidity data were processed into annual and seasonal averages. The distance to three main industries (two multinational refineries and one multinational pulp and paper manufacturer) was measured and the percentage time the wind blows from the direction of those industries to a receptor.

The selection of buffer radii was based on previous ESCAPE studies [25]. Buffers of 50, 100, 300, 500, and 1000 m for major and minor road length and 1000 m for distance to major and minor roads to account for background emissions of NO_2_, related to traffic emissions were defined. Buffer distances of 100, 300, 500, 1000, and 2000 m were defined for all other variables. Each buffer was used to intersect the different predictor variables to allow for points, lengths, and areas to be calculated. The predictors used for the LUR models, buffer size, rational for inclusion, as well as expected direction of effect are presented in the supplementary material Table S1.

### 2.4. Land Use Regression Modelling

Using the ESCAPE [19,25] approach, standard linear regression was used to develop land use regression models to predict air pollutant concentrations. The model that yields the highest percentage explained variability (R^2^) and minimizes the error (root mean square, RMSE) was selected for use. To develop a regression model for each pollutant, a forward stepwise procedure was followed.

Model validation was undertaken using the leave-one-out cross validation (LOOCV) method. The model was developed for *n* − 1 sites and the predicted concentrations compared to the measured concentration at the left-out site. This process was undertaken *N* times and the relationship between the predicted and observed concentrations, across all sites, then computed as a measure of model performance.

All modelling was performed using the Statistical Package STATA version 15 (StataCorp LLC., College Station, TX, USA). 53 variables were regressed individually against the NO_2,_ SO_2_, PM_10_, and PM_2.5_ concentrations for each season as well as an annual average for this assessment. As such, a total 12 LUR models were developed.

## 3. Results

### 3.1. Air Pollutant Measurements

Overall, 90 SO_2_, 100 NO_2_, 51 PM_10_ and PM_2.5_ measurements were taken over the duration of the 1-year monitoring period. Intermittent air pollution measurements at the monitoring points were adjusted in line with monitoring data from the reference site to provide annual averages. A high correlation was observed for the duplicate NO_2_ (R^2^ = 0.92), SO_2_ (R^2^ = 0.85), and PM_10_ (R^2^ = 0.99) samplers over the 1-year monitoring period at the reference site. No duplicate samples were collected for PM_2.5_. Adjusted average annual as well as seasonal NO_2_, SO_2_, PM_10_, and PM_2.5_ concentrations measured across both north Durban and south Durban are presented in Table 1 below. The N refers to the number of monitoring points. There were initially 20 PM and 40 NO_2_/SO_2_ monitoring sites, however points at which outlier measurements were recorded at/or samplers were stolen were completely removed from the dataset.

Box plots of the annual and seasonal concentrations measured in north Durban and south Durban for each pollutant are presented in supplementary material (Figure S1).

### 3.2. Land Use Regression Models

In the NO_2_ models (Table 2), traffic and industrial variables emerged as predictors in expected directions, as expected across the seasons and in the annual model. Of particular interest was the role of the Harbor variable, which was a significant predictor in the summer and annual models. This variable was not a predictor in the modelling of the other pollutants.

Traffic and industrial variables were, as expected, important predictors across the majority of pollutants. Specific traffic variables varied across the pollutants, with road length serving as a proxy for the PM_10_ and the summer PM_2.5_ models, while the LDMV variable emerged as significant for the SO_2_ and PM_2.5_ models. Indicative of the geography of Durban, elevation was a negative predictor in the NO_2_ and PM_10_ models. However, elevation was not a predictor in the other models (see also Table 3, Table 4 and Table 5).

Population emerged as a predictor in only the winter NO_2_, summer PM_10_, and annual PM_2.5_ models. Other urban predictors, such as urban space, were significant in the winter models of PM_10_ and PM_2.5_.

In all the pollutant models (annual, summer, and winter) VIF was considered reasonable (<5) suggesting that collinearity was not an issue in these models. Furthermore, the *p*-value was less than 0.1 for each coefficient suggesting that they were statistically significant [25] predictor variables.

The SO_2_, NO_2_, PM_10_, and PM_2.5_ annual and seasonal LUR models were set for the north and south areas, respectively, to identify potential predictors of these pollutants. The area between Durban north and Durban south can be characterized by areas of open spaces, and an increase in topography (height above sea level) as distance from the coastline increases. A limited amount of urban land use is found in this area away from the city center and, furthermore, no industrial activity occurs in this area. As a result, the area between Durban north and Durban south will most likely have a negligible influence on the air pollution on each of these localities.

## 4. Discussion

A key finding in the development of exposure models for the city of Durban was the influence of the Harbor variable in our models. This was particularly true for the annual and summer NO_2_ LUR models suggesting that the Harbor and its associated activity may be an influential source of pollution. Durban is the busiest port in Africa and has the second largest container terminal in the southern hemisphere. For the ease of transporting goods and other commodities to and from the Harbor, major industries in the region developed around the port. To transport goods for import and/or export purposes, the South African National Roads Agency Limited Ltd (SANRAL, Pretoria, South Africa) developed an advanced road network to service the inland cities of the country. As a result, air pollution is not only emitted from industries in the DSB, but also from vehicles that transport goods to and from the port [26].

A recent study demonstrated that the total annual NO_2_ emissions from ships in the Durban Port were calculated at 1116.87 tonnes per annum and were slightly lower than two petrochemical refineries emissions of 1760 and 1241 tonnes per annum in 2010 and 2011, respectively [27]. These results show that the port emissions should not be ignored in cumulative air quality impact assessments. Furthermore, the study shows a higher number of vessels frequenting the terminal between May and September, suggesting reason for the Harbor variable being present in the winter NO_2_ LUR model.

A study making use of the operational meteorological air quality model (OML) to calculate the urban dispersion of air pollutants originating from ships in three Danish ports, Copenhagen, Elsinore, and Koge, showed that oxides of nitrogen (NO_x_) emitted by ships in the port of Copenhagen and Elsinore contributed substantially to the overall NO_x_ pollution in their respective areas [28]. Furthermore, in LUR models estimating air pollution exposure of NO_2_ and NO_x_ in 36 study areas in Europe, the Harbor variable was statistically significant in models for other port cities such as Copenhagen, (Denmark), Ruhr Area, (Germany), and Heraklion (Crete) [16].

The monitoring results confirmed a general increase in NO_2_, PM_10_, PM_2.5_, and SO_2_ concentrations during winter and a decrease in NO_2_, PM_10_, PM_2.5_, and SO_2_ concentrations during summer. The results measured during spring are similar to the annual average and are lower than those measured during winter and higher than those measured during summer. The nature and characteristics of air pollution dispersion over Durban is known to fluctuate during the year with the change of the seasons [29]. During winter, the slow moving South Indian High is responsible for clear skies, low levels of precipitation, and weak north-easterly winds. The frequency and strength of inversions are known to be greatest during winter months of June and July. Surface inversions trap air pollution by inhibiting adequate air pollution dispersion.

In a greater volume of atmosphere, the ability for air pollutants to disperse increases and there is a greater chance for its concentration to be reduced. During summer, mainly unstable conditions are found to develop, and the depth of the mixing layer is increased thereby enabling free convection of air into the upper boundary layer. This phenomenon assists air pollutants to disperse into the upper atmosphere. Conversely, during winter, particularly in the early mornings, stable conditions arise, and the vertical diffusion of air pollutants is limited. A surface inversion may exist nearer to the ground, or pollutants may be diffused upward only to be halted by an elevated inversion layer. Both of these surface and elevated inversions need to be considered in the DSB when analyzing their relationship with air pollution dispersion.

From a spatial distribution perspective, measured SO_2_ and NO_2_ concentrations (annual and seasonal averages) were higher at the south Durban sites than at the north Durban sites. Key industries in the DSIB include two major oil refineries, owned by multinational corporations, a multinational paper and pulp plant, a sugar mill, several chemical industries, the port and some 600 other smaller industries. Measured PM_10_ and PM_2.5_ concentrations (annual and seasonal) were on average higher at the north Durban sites than the south Durban sites. Besides industrial or vehicular emitters other possible sources for the high PM concentrations identified in the north are large open fields, regular field fires, sugar cane fires, and domestic burning of garden and other refuse, plus major earthworks and construction [30].

In our study, the concentrations of ambient PM_10_ and PM_2.5_, NO_2_, and SO_2_ throughout the mixed industrial and residential land use types in eThekwini and the subsequent LUR modelling present interesting variation across the intra-city regions for the different pollutants. Although the study included a heavy industrialized area, the industry variable did not emerge strongly across all pollutant LUR models. Traffic variables were consistently statistically significant in all LUR models, and this may imply that this type of modelling works well for air pollution sources that are localized in extent [31]. Total length major road, distance to minor roads, total number of motor vehicles, urban land use type, and population, returned positive correlations with PM_10_, PM_2.5_, and NO_2_ concentrations while open space and elevation returned negative correlations. In all (annual and seasonal) models, PM_10_, PM_2.5_, and NO_2_ levels were influenced by traffic variables as well as population and has this been demonstrated with other studies [13,32].

Population density (an indication of domestic fuel burning) was present in the winter NO_2_, summer PM_10_, as well as annual PM_2.5_ LUR models. This is consistent with the findings reported in other studies [4,16,33], in which the population density variable has been related to these sources of PM. While it was expected for a traffic variable to have appeared in the winter NO_2_ model, the population variable appeared instead, indicating that areas with higher population density are related to higher NO_2_ levels during winter.

The open space and elevation variables were seen as having a negative influence in the NO_2_, PM_10_, and PM_2.5_ concentrations. An increase in open space (areas such as urban forests and parks) can reduce local NO_2_, PM_10_, and PM_2.5_ concentrations and improve respiratory health in the area and has empirically been shown in other studies [34,35,36].

The importance of considering elevation as an influential factor for coastal cities has been highlighted by [37,38]. Elevation exhibited a negative interaction with annual average NO_2_ and PM_10_ as well as winter NO_2_ and PM_10_ concentrations indicating that elevated areas (i.e., outside of south Durban) had the lowest NO_2_ and PM_10_ levels.

The SO_2_ LUR models were similar to the NO_2_ LUR models in that the industrial land use coverage variable as well as the number of LDMV variables were retained in all annual and season models. In a study undertaken by [39], the highest SO_2_ values were also associated with industrial and traffic emission sources. Industrial emissions associated with stacks that are released at a height, impact the ground level at a distance away from the source. For very fine scale concentrations in and around the industrial area, dispersion models may be useful to supplement this model; however, these models require emission data. The poor strength of the LUR models developed for SO_2_, however, suggests that the industrial land use variables need to be carefully considered when being applied in a LUR assessment. However, our results agree with the other SO_2_ LUR studies that R^2^ values for SO_2_ tend to be suppressed [40].

There were no input variables of Harbor in the SO_2_ LUR model. A recent study which calculated the pollution from ships in the Durban Port found that SO_2_ emissions from ships that frequent the port are far less than that emitted from just two large petrochemical refineries and a paper mill operating nearby [27]. Therefore, while SO_2_ is emitted during ship hoteling at the port, the SO_2_ emissions from industries located near the port far outweigh the SO_2_ emissions being released from the ships hoteling at the Harbor. Furthermore, in a study making use of an operational meteorological air quality model (OML) to calculate the urban dispersion of air pollutants originating from ships in three Danish ports, Copenhagen, Elsinore, and Koge, ships in Copenhagen and Elsinore (both Harbors) contribute insignificantly to urban pollution with respect to SO_2_ [28]. In Koge, however, the low activity in the Harbor meant that ships did not significantly affect urban air quality.

In the annual and winter PM_10_ models as well as summer PM_2.5_ models, total length major roads was seen as a significant predictor variable. This likely reflects the major impact of motorized road traffic emissions as well as road dust being suspended from tire and break wear and tear on PM levels [19]. Furthermore, In South Africa, a substantial fraction of private cars and most middle and heavy-duty vehicles make use of diesel as fuel for economic reasons. Other sources of PM such as large open fields, regular field fires, etc., were not taken into account in the LUR model due to limited data due a limited number of PM sampling equipment used in this study.

While a study undertaken by [41] confirms that sea spray does contribute to PM levels at coastal cities, distance to coastline was not regarded as insignificant predictor variable in the results. The Bluff Ridge between the ocean and DSIB which acts as a buffer to sea spray to samplers located in the south Durban could be attributed to PM having a poor relationship with distance to coastline. In north Durban, samplers were located from approximately 2 km inland of the ocean. Sea spray is known to decrease with increasing distance away from the shoreline [42].

To determine the effects of change in wind regime during each season, the inclusion of wind direction as a meteorological variable, which assessed the percentage time wind blows from the two refineries and the paper mill towards each monitoring point, was assessed. The inclusion of wind direction in relation to direction of industry however was not regarded as a significant predictor variable in this study. Furthermore, the inclusion of annual and seasonal average wind speed, ambient temperature, and humidity also did not emerge as significant variables.

A similar LUR study was undertaken to explain the spatial variation of ambient NO_x_ concentrations in Durban south [43]. In this study, two 2-week NO_x_ monitoring campaigns were undertaken during summer and winter and an adjusted annual average was used for the model development. Our study serves to build onto the work already undertaken by Muttoo et al. (2016) [43] by developing annual and seasonal LUR models for additional air pollutants (e.g., NO_2_, SO_2_, PM_10_, and PM_2.5_) across Durban north and Durban south areas. This was achieved by undertaking a more comprehensive air pollution monitoring campaign for three periods of 2 weeks per a site in the cold, warm, and one intermediate temperature season between July 2015 and March 2016 as well as taking measurements at one additional site (reference site) for 1 year. Furthermore, our study was the first to explore the effect of meteorology on measured pollutant concentrations, in South Africa, given the unique topographical domain of our study area. The key similarity in the findings of the annual NO_x_ model developed by Muttoo et al. (2016) [43] and our annual NO_2_ model was the presence of the traffic variable, suggesting a strong influence of traffic on NO_x_ related components.

## 5. Conclusions

In conclusion, while findings of the study highlighted the importance of traffic and land use type (more specifically, industry) variables in influencing air pollutant concentrations, interestingly the Harbor variable also emerged as a significant predictor. It is acknowledged that a key limitation of the study is the sample size used to develop the LUR models especially for PM due to restricted resources available. Furthermore, the inability of the model to consistently identify industrial land use also suggests a modelling limitation. The LUR model may not be the ideal tool when estimating industrial emissions from stacks as the resultant plume would only reach ground level some distance away from the source. A further improvement of the LUR models could include predictor variables that characterize industrial emissions in order to capture local variations due to emission patterns [44]. An air pollution dispersion model, which incorporates meteorological data at a high resolution (example hourly), terrain, physical, and chemical characteristics of the air pollution is recommended to simulate the formation and transport of the plume, suggesting the need for hybrid modelling [45].

## Figures and Tables

**Table 1:** Adjusted annual average annual and seasonal nitrogen dioxide (NO_2_), sulphur dioxide (SO_2_), PM_10_, and PM_2.5_ concentrations (µg/m^3^).

Pollutant	Season	Mean	Standard Deviation	Minimum	Maximum
**NO_2_**	Annual average	17.0	3.9	6.5	24.0
	Summer average	10.5	2.8	4.1	17.3
	Winter average	25.8	6.7	10.1	42.3
	Spring Average	20.4	5.1	7.6	29.5
**SO_2_**	Annual average	3.4	1.6	1.5	7.8
	Summer average	2.8	1.3	0.7	6.4
	Winter average	4.2	1.9	1.8	9.2
	Spring average	3.3	1.5	1.4	7.4
**PM_10_**	Annual average	36.6	19.2	11.0	99.7
	Summer average	20.5	10.0	9.3	54.1
	Winter average	50.3	27.0	15.2	138.1
	Spring average	38.5	21.9	8.91	107.7
**PM_2.5_**	Annual average	12.3	5.7	3.2	31.0
	Summer average	8.5	4.0	2.2	21.5
	Winter average	17.0	8.0	4.5	43.1
	Spring average	11.4	5.3	3.1	29.4

**Table 2:** Annual, summer, and winter NO_2_ land use regression (LUR) model results.

Season	Predictors	Unit	R^2^	LOOCV	df	Beta	Standard Error	t	*p*
Annual	Intercept	-	0.6	0.4	32	1.85 × 10^1^	2.16 × 10^0^	24.5	0.0
	Total length major roads (100 m)	m				2.07 × 10^−2^	1.12 × 10^−1^	4.4	0.0
	Harbor (2000 m)	m				4.32 × 10^−7^	4.50 × 10^−3^	1.9	0.0
	Elevation	m				−3.68 × 10^−2^	2.24 × 10^−7^	−3.6	0.0
Summer	Intercept	-	0.4	0.2	32	8.32 × 10^0^	6.43 × 10^−1^	12.9	0.0
	Distance to minor roads	m				5.66 × 10^−2^	1.71 × 10^−2^	3.3	0.0
	Industrial (1000 m)	m				1.98 × 10^−6^	8.79 × 10^−7^	2.3	0.0
	Harbor (2000 m)	m				4.97 × 10^−7^	2.30 × 10^−7^	2.2	0.0
Winter	Intercept	-	0.6	0.5	30	2.44 × 10^1^	1.43 × 10^0^	17.1	0.0
	Elevation	m				−5.25 × 10^−2^	1.58 × 10^−2^	−3.3	0.0
	Population (1000 m)	m				8.25 × 10^−4^	1.91 × 10^−4^	4.3	0.0
	Industrial (100 m)	m				3.75 × 10^−4^	1.61 × 10^−4^	2.3	0.0

**Table 3:** Annual, summer, and winter SO_2_ LUR model results.

Season	Predictors	Unit	R^2^	LOOCV	df	Beta	Standard Error	t	*p*
Annual	Intercept	-	0.4	0.2	37	2.5 × 10^0^	3.0 × 10^−1^	8.4	0.0
	Industrial (500 m)	m				7.9 × 10^−6^	1.9 × 10^−6^	4.1	0.0
	Total number LDMV (100 m)	No				8.4 × 10^−8^	3.1 × 10^−8^	2.8	0.0
Summer	Intercept	-	0.5	0.3	29	1.4 × 10^0^	3.1 × 10^−1^	4.5	0.0
	Industrial (2000 m)	m				4.1 × 10^−7^	9.9 × 10^−8^	4.1	0.0
	Total number LDMV (100 m)	No				7.1 × 10^−8^	2.2 × 10^−8^	3.2	0.0
Winter	Intercept	-	0.5	0.4	29	2.6 × 10^0^	5.7 × 10^−1^	4.6	0.0
	Industrial (2000 m)	m				5.9 × 10^−7^	1.9 × 10^−7^	3.1	0.0
	Total number LDMV (300 m)	No				3.5 × 10^−8^	1.7 × 10^−8^	2.1	0.0

**Table 4:** Annual, summer, and winter PM_10_ LUR model results.

Season	Predictors	Unit	R^2^	LOOCV	df	Beta	Standard Error	t	*p*
Annual	Intercept	-	0.8	0.7	14	3.2 × 10^1^	2.2 × 10^0^	14.0	0.0
	Total length major road (1000 m)	m				5.3 × 10^−3^	8.8 × 10^−4^	6.0	0.0
	Elevation	m				−1.1 × 10^−1^	4.4 × 10^−2^	−2.4	0.0
Summer	Intercept	-	0.5	0.2	13	1.2 × 10^1^	2.4 × 10^0^	5.1	0.0
	Population (2000 m)	m				2.3 × 10^−4^	7.1 × 10^−5^	3.3	0.0
	Total number HDMV (100 m)	No				8.8 × 10^−6^	4.2 × 10^−6^	2.1	0.0
Winter	Intercept	-	0.8	0.6	13	2.5 × 10^1^	9.7 × 10^0^	2.6	0.0
	Total length major road (1000 m)	m				4.4 × 10^−3^	1.8 × 10^−3^	2.5	0.0
	Elevation	m				−1.9 × 10^−1^	6.5 × 10^−2^	−3.0	0.0
	Urban (2000 m)	m				4.0 × 10^−6^	2.0 × 10^−6^	2.0	0.0

**Table 5:** Annual, summer, and winter PM_2.5_ LUR model results.

Season	Predictors	Unit	R^2^	LOOCV	df	Beta	Standard Error	t	*p*
Annual	Intercept	-	0.8	0.6	13	1.1 × 10^1^	7.9 × 10^−1^	14.0	0.0
	Open space (100 m)	m				−2.2 × 10^−4^	7.2 × 10^−5^	−3.1	0.0
	Total number LDMV (100 m)	No				1.8 × 10^−7^	5.6 × 10^−8^	3.2	0.0
	Population (2000 m)	m				5.3 × 10^−5^	2.3 × 10^−5^	2.4	0.0
Summer	Intercept	-	0.7	0.7	15	7.6 × 10^0^	5.9 × 10^−1^	13.0	0.0
	Total length major road (500 m)	m				2.4 × 10^−3^	4.0 × 10^−4^	6.0	0.0
Winter	Intercept	-	0.6	0.6	14	−8.9 × 10^0^	4.8 × 10^0^	−0.19	0.0
	Total number LDMV (100 m)	No				2.5 × 10^−7^	1.3 × 10^−7^	2.0	0.0
	Urban (100 m)	m				5.7 × 10^−4^	1.8 × 10^−4^	3.2	0.0

## Supplementary Materials

The following are available online at https://www.mdpi.com/1660-4601/17/15/5406/s1, Figure S1: Distribution of NO_2_, SO_2_, PM_10_, PM_2.5_ annual and seasonal means for Durban north and Durban south. Median, interquartile range (IQR), and whiskers present, Table S1: Variables used, buffer radius, rational for inclusion, and expected direction of effect.
