# Supplementary material for: Harbor and Intra-City Drivers of Air Pollution: Findings from a Land Use Regression Model, Durban, South Africa
Source: Int J Environ Res Public Health. 2020 Jul 27;17(15):5406. doi: 10.3390/ijerph17155406 (PMC7432936; doi:10.3390/ijerph17155406)
Supplement: Supplementary file 1 [file ijerph-17-05406-s001.pdf]

Table S1. Variables used, buffer radius, rational for inclusion and expect direction of effect.

Variable Category	Variables (unit)	Buffer Radius (m)	Rational for Inclusion	Expected Effect
Road length	Length of major road (m)	50, 100, 300, 500, 1000	Road length is associated with air pollutant emissions.	+
	Length of minor road (m)			+
Distance to road	Distance to major road (m)	1000	Distance to roads is associated with air pollutant emissions.	+
	Distance to minor road (m)			+
Traffic Intensity	Number of LMV	100, 300, 500, 1000, 2000	Traffic intensity is an indication of vehicle emissions.	+
	Number of HMV	100, 300, 500, 1000, 2000		+
Land use	Area of industrial (m^2)	100, 300, 500, 1000, 2000	Land use type is a function of air pollution emission.	+
	Area of open space (m^2)			-
	Area of urban (m^2)			+
	Area of harbour (water) (m^2)			+
Population density	Population density	100, 300, 500, 1000, 2000	Pollution from human activity.	+
Physical geography	Altitude (m)	N/A	Pollutant levels are higher at ground level and tend to decrease with increasing height.	-
	Distance to coastline (m)	N/A	Land/sea breeze interaction can lead to effective air pollution dispersion.	-
Meteorology	Percentage time wind blows from SAPREF (%)	N/A	Wind blowing from the direction of key industries can affect air pollution concentrations.	+
	Percentage time wind blows from Engen (%)	N/A		+
	Percentage time wind blows from Mondi (%)	N/A		+
	Ambient temperature (°C)	N/A	Increased temperature results in improved dispersion of pollution.	-
	Wind speed (m/s)	N/A	Increased wind speed results in improved dispersion of pollution.	-
	Humidity (%)	N/A	Increased humidity results in improved dispersion of pollution.	-
Regional Variable	Regional variation in air pollution concentrations	N/A	Pollutant levels in north Durban are lower than in south Durban.	-

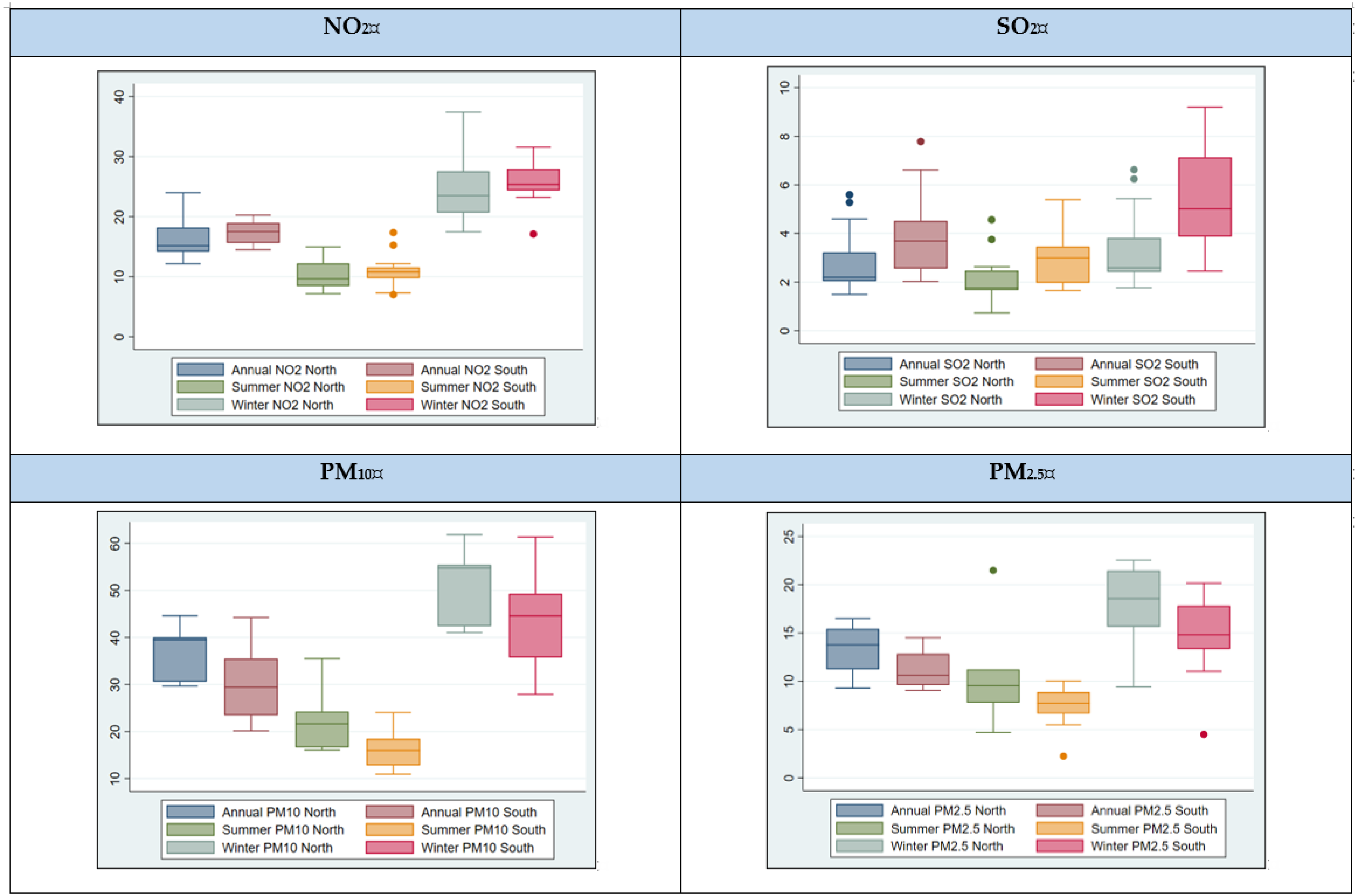

Figure S1. Distribution of NO_2, SO_2, PM_{10}, $PM_{2.5}$ annual and seasonal means for Durban north and Durban south. Median, interquartile range (IQR) and whiskers present.

Quality Assurance/Quality Control Procedures for Sampling Protocol

Internal Quality Control

1. Document consistency of rotameters by regular comparison with the same internal standard. The internal standard can be a soap film meter, a dry gas meter or a rotameter that remains in the laboratory all the time. Record the readings in a table. The comparison must be performed before and after the study period and every three months after the start of the study.
2. Quality control procedures for weighing and reflectance measurement are in the SOPs.
3. Collect a field blank every measurement period so that 12 field blanks will be obtained. Take filter to the field, load the sampler with the filter, leave in the field for two weeks and take filter to the lab. Use the continuous sampling site for blanks. Use a PM_{10} impactor that is idle during the week. We will not take $PM_{2.5}$ blanks. The blanks are used to determine the detection limit and average field blank.
4. Conduct measurements with two collocated PM_{10} samplers every measurement period (12 sampling days). Field duplicates are taken to document precision of the measurements. Duplicates will be obtained at the continuous reference site.

External Quality Control

1. During a site visit to all PM monitoring points a comparison will be made of the used rotameters with one certified standard flow measurement device.
2. The impact of transporting filters on weighing and reflectance measurements will be checked by a control program that will involve analysis of five freshly exposed and five blank filters before and after transport to one of the centers involved.
